# Supplementary material for: Ferritin Metabolism Reflects Multiple Myeloma Microenvironment and Predicts Patient Outcome
Source: Int J Mol Sci. 2023 May 16;24(10):8852. doi: 10.3390/ijms24108852 (PMC10218632; doi:10.3390/ijms24108852)
Supplement: Supplementary file 1 [file ijms-24-08852-s001.zip › ijms-2235145-supplementary.pdf]

Supplementary Materials

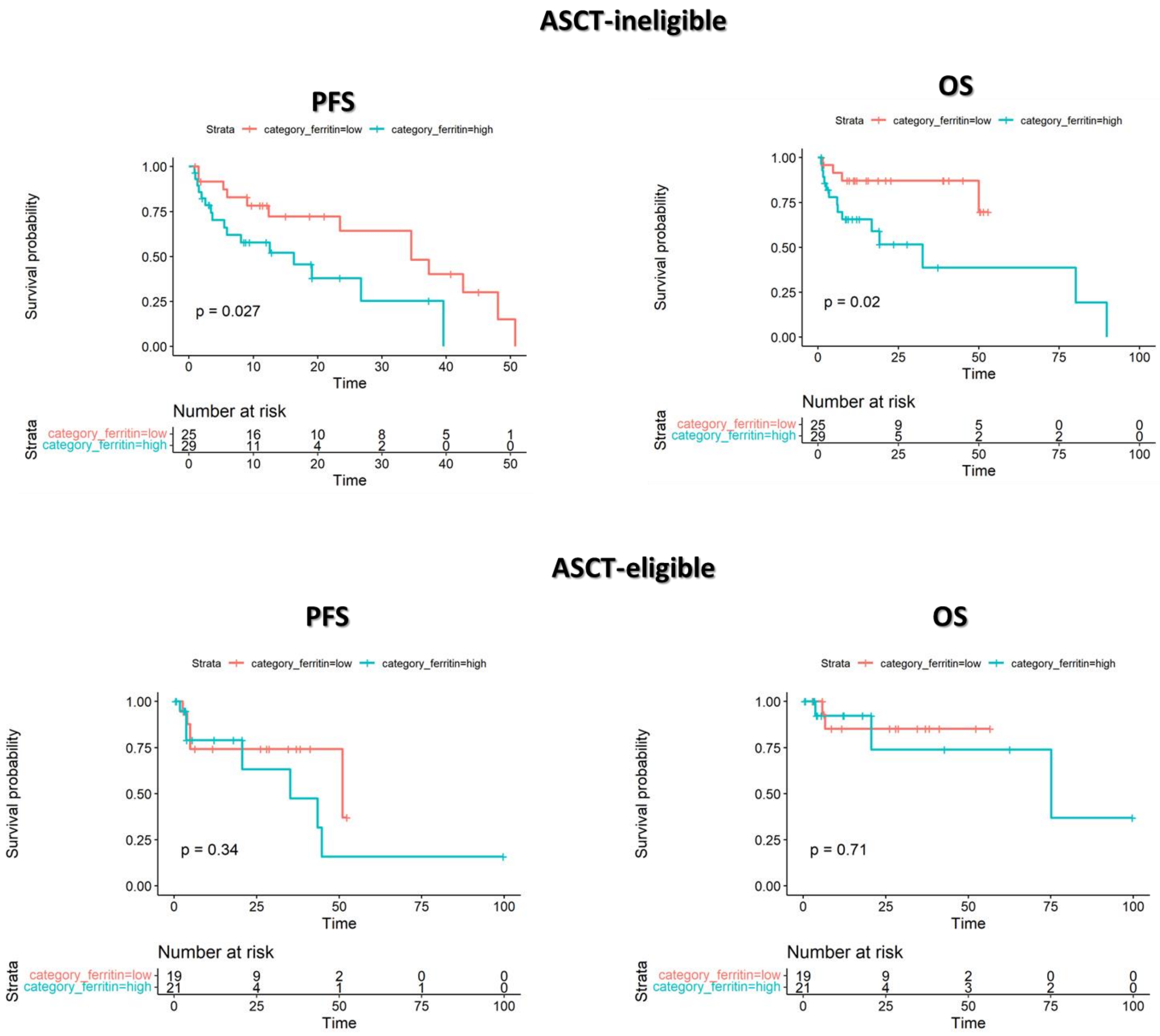

Supplementary Figure S1: survival according to ASCT eligibility and ferritin values

PFS and OS results for MM patients according to their transplant eligibility status. Please note that a significant value at the log-rank test was reached for ASCT-inelegible patients only.

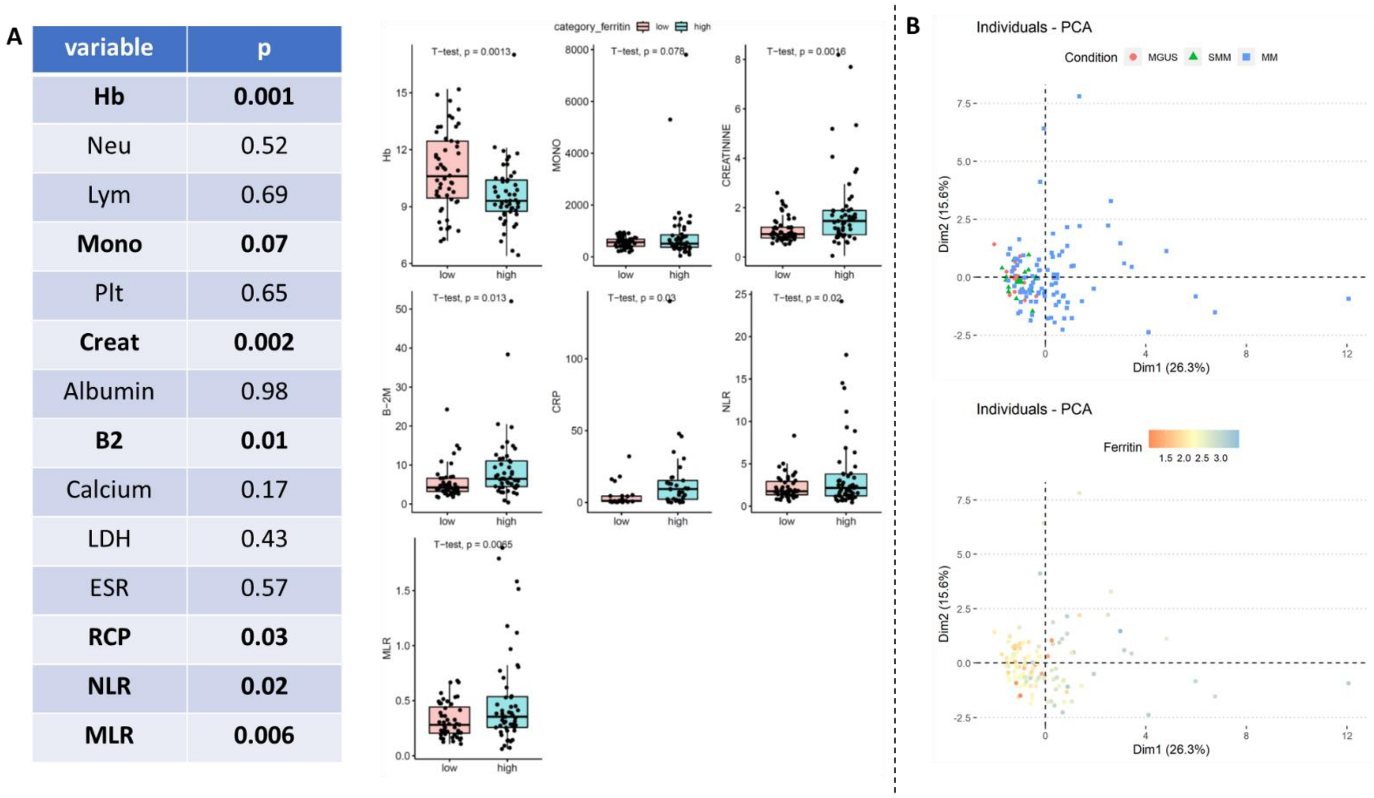

variable	p
**Hb**	**0.001**
Neu	0.52
Lym	0.69
**Mono**	**0.07**
Plt	0.65
**Creat**	**0.002**
Albumin	0.98
**B2**	**0.01**
Calcium	0.17
LDH	0.43
ESR	0.57
**RCP**	**0.03**
**NLR**	**0.02**
**MLR**	**0.006**

Supplementary Figure S2: correlation of laboratory variables with high/low levels of ferritin

A. Left: list of all variables tested for differences between high and low level ferritin groups and their p values according to t-Student test. On the right boxplots showing the results of the variable reaching a statistical significance. **B.** principal component analysis performed on significant values in MGUS, SMM and MM patients. Interestingly, all MGUS and SMM clusterize closer to the group of low ferritin MM patients.

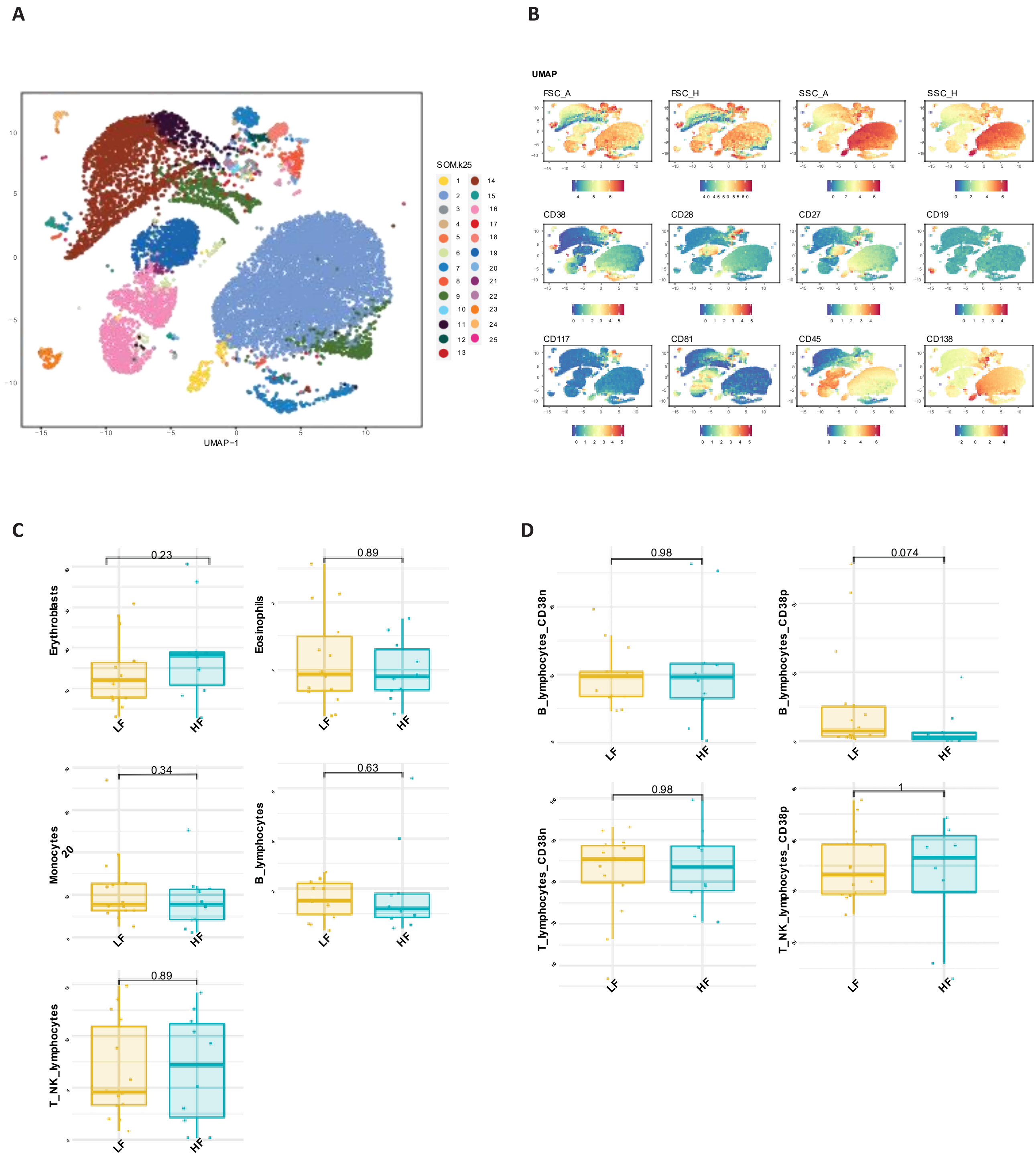

Supplementary Figure S3: FlowCT analysis of BD PCD tube

(A) Uniform manifold approximation and projection (UMAP) of 25 clusters identified by self-organizing map (SOM) of BD OneFlow™ PCD tube. (B) Median expression of individual marker in each cell cluster identified by SOM represented in multi-UMAP plot. (C) Boxplots showed the different frequency of eosinophils, erythroblasts, B and T lymphocytes, and monocytes between low ferritin level group (LF) and high ferritin level group (HF). (D) Boxplots showed the different frequency of T and B lymphocyte subsets between low ferritin level group (LF) and high ferritin level group (HF).

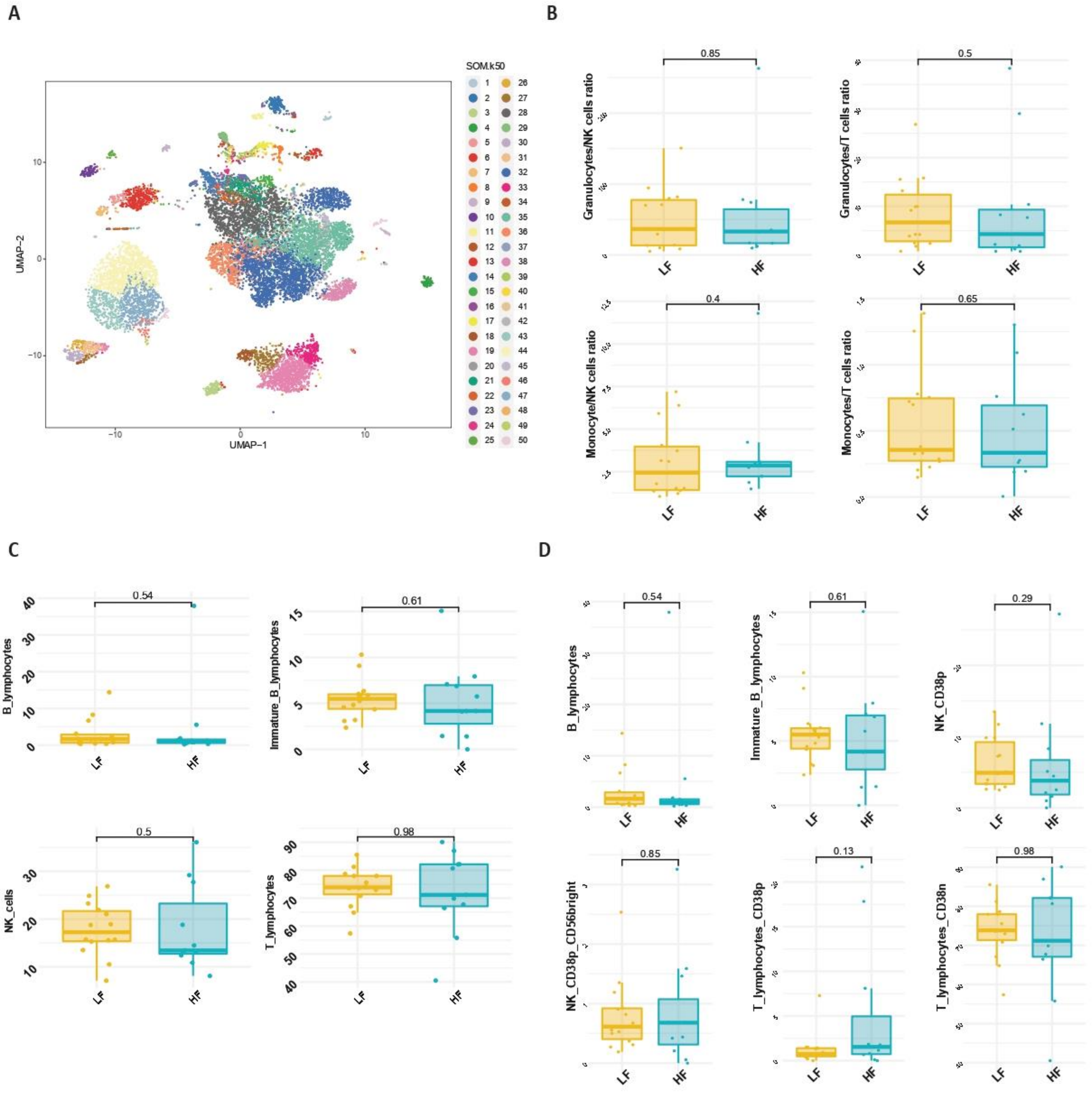

Supplementary Figure S4: FlowCT analysis of PCST tube

(A) Uniform manifold approximation and projection (UMAP) of 50 clusters identified by self-organizing map (SOM) of BD OneFlow™ PCST tube. (B, C) Boxplots showed the different frequency and ratio (with granulocytes and monocytes) of T and B lymphocytes and NK cells between low ferritin level group (LF) and high ferritin level group (HF). (D) Boxplots showed the different frequency of T and B lymphocyte and NK cell subsets between low ferritin level group (LF) and high ferritin level group (HF) after lymphocytes subclustering.

A

Term ID	Related Component
GO:0070288	ferritin complex
GO:0070289	extracellular ferritin complex
GO:0008043	intracellular ferritin complex

Final gene list

FTH1	GINS2	HBB	STEAP3	STXBP2
FTL	GINS3	HFE	MCM10	TFR2
CSDE1	GINS4	HMOX1	PRF1	TTC26
PELO	LYST	XIAP	SLC30A10	PUS1
CNOT6	CP	IFNG	NLRC4	HAVCR2
DRD4	CPOX	ITK	BCS1L	STX11
DRD3	HJV	KCNN4	BMP2	SLC7A7
DRD2	UNC13D	GLRX5	SLC4A1	PIEZO1
KCNH2	GFER	PKLR	SLC19A1	HAMP
GINS1	SLC40A1	SLC25A38	STAT2	

B

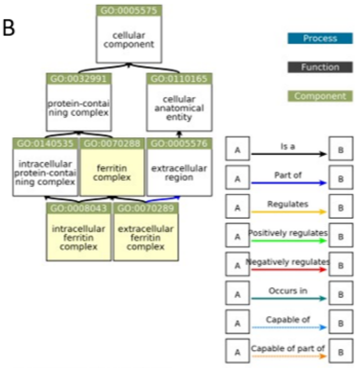

C

Number of Genes	Category	Description	p-value	FDR value	transferred FDR value
25	GO Biological Process	Response to stress	1.38E-07	8.06E-05	0.409366496
19	GO Biological Process	Ion transport	2.21E-10	2.84E-07	0.654668166
9	GO Biological Process	Cellular iron ion homeostasis	1.93E-13	8.28E-10	0.908196966
7	STRING Clusters	Mixed, incl. hemochromatosis, and ferritin complex	2.46E-13	2.80E-10	0.955284197
6	COMPARTMENTS	Ferritin complex	9.33E-12	2.16E-08	0.766554625
6	WikiPathways	Ferroptosis	1.54E-09	5.21E-07	0.628316228
6	KEGG Pathways	Ferroptosis	1.76E-09	5.91E-07	0.622841252
3	COMPARTMENTS	Intracellular ferritin complex	2.91E-07	1.30E-04	0.388605665
3	COMPARTMENTS	Extracellular ferritin complex	1.74E-06	5.70E-04	0.324412514
2	GO Cellular Component	Intracellular ferritin complex	3.66E-05	0.0124	0.190657831
2	UniProt Keywords	Iron storage	1.30E-04	0.0088	0.205551733

Supplementary Figure S5: a ferritin metabolism-related gene-set that contains 49 genes highly correlated with ferritin biosynthesis : A: 3 main gene ontology term was used as a fundamental framework for gene dataset construction. Additionally, we add another dataset from Garcia-Casal MN et all (PMID:29723227) from Molecular Signatures Database (MSigDB) to create a dataset that has 49 unique genes. **B:** GO domains network representation. **C:** Enrichment in GO terms from biological processes and KEGG pathways for the identified genes clearly revealed considerable enrichment in ferritin metabolism-related terms, with p-values<0.005 and FDR values< 0.001 regarded as significant enrichment scores.

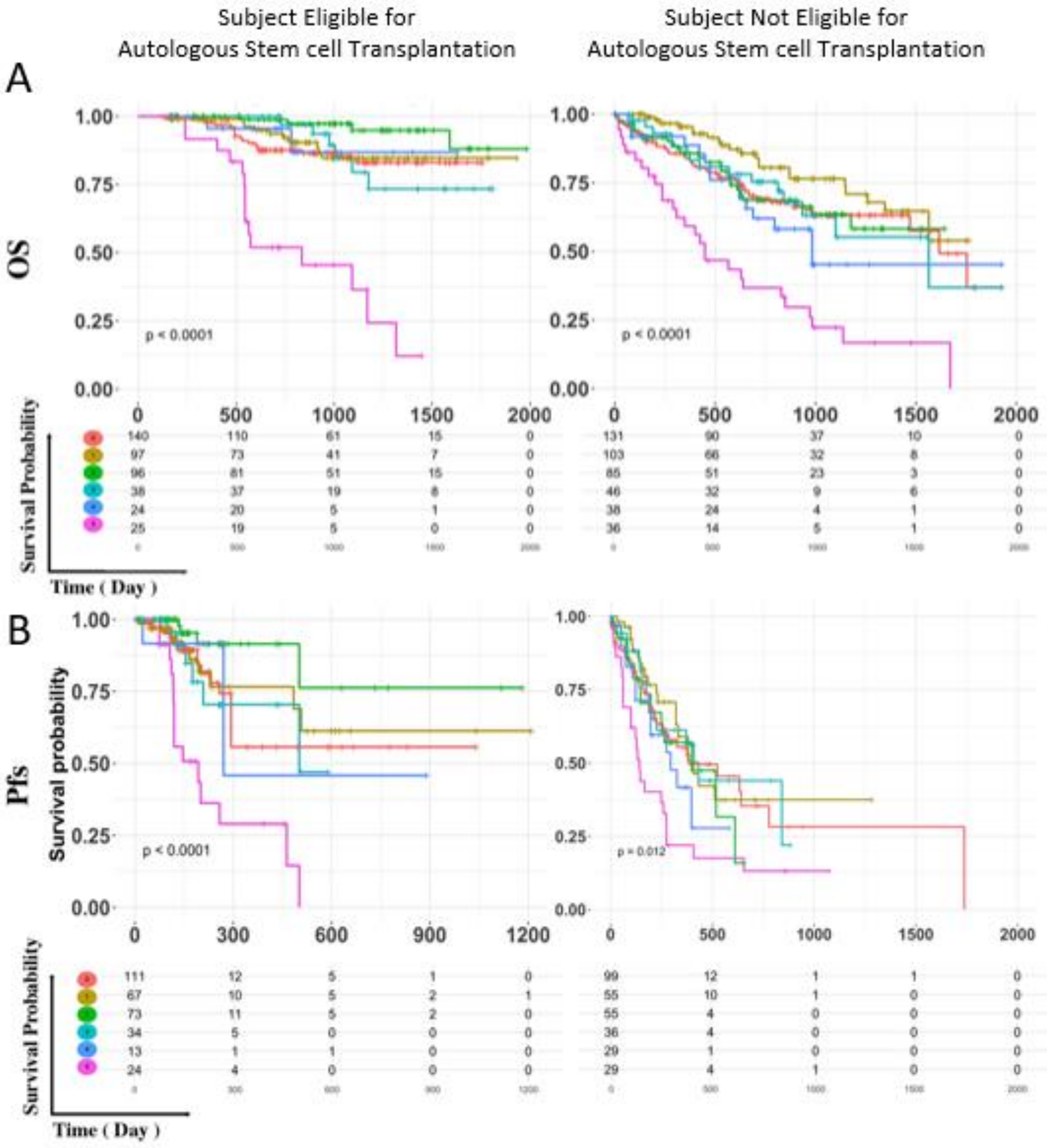

Supplementary Figure S6: Survival analysis of unsupervised patients clusters: based on different ferritin related and/or conserved diverse biological Functions, six different clusters have been identified. Patients were further divided according to their ASCT eligibility: overall survival (OS) and progression free survival (PFS) results were reported in A and B respectively. For both panels, the left picture indicates subjects who are eligible for the ASCT while the right picture subject who are not eligible for ASCT.

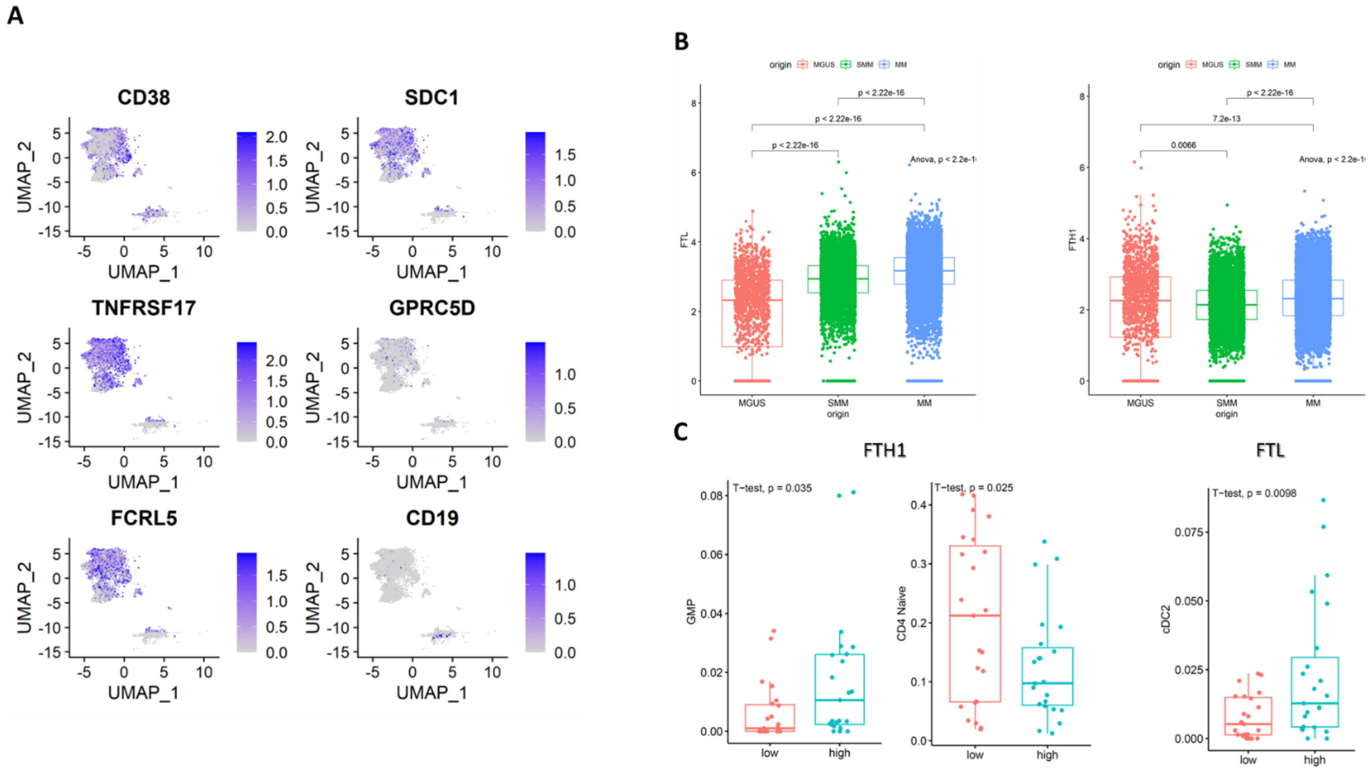

Supplementary Figure S7: single cell RNAseq results: A. gene expression of main markers of plasma cells. B. FTL and FTH1 expression in different conditions and their comparative and overall p.values. C. Additional significant changes in immune populations associated to high or low levels of FTH1/FTL

Supplementary Table S1. Main characteristics of MGUS and SMM patients included in the study

	MGUS (15)	SMM (17)
Sex (M/F)	9/6	8/9
Hb (g/dl)	13.3	12.9
Neutrophils (µL)	4200	4474
Lymphocytes (µL)	2152	1927
Monocytes (µL)	499	482
Platelets (µL)	245333	250725
Creatinin (mg/dL)	0.98	0.86
Albumin (g/L)	6.85	3.97
Calcium (mg/dL)	9.49	9.65
β2M (mg/dL)	2.95	2.68
LDH (U/L)	160	151
Ferritin (ng/ml)	139	116
